# Pharmacological intervention of the FGF–PTH axis as a potential therapeutic for craniofacial ciliopathies

**DOI:** 10.1242/dmm.049611

**Published:** 2022-08-16

**Authors:** Christian Louis Bonatto Paese, Ching-Fang Chang, Daniela Kristeková, Samantha A. Brugmann

**Affiliations:** 1 Division of Developmental Biology, Cincinnati Children's Hospital Medical Center, Cincinnati, OH 45229, USA; 2 Department of Pediatrics, University of Cincinnati College of Medicine, Cincinnati, OH 45229, USA; 3 Laboratory of Molecular Morphogenesis, Institute of Animal Physiology and Genetics, v.v.i., Czech Academy of Sciences, Brno 602 00, Czech Republic; 4 Department of Experimental Biology, Faculty of Science, Masaryk University, Brno 625 00, Czech Republic; 5 Division of Plastic Surgery, Department of Surgery, Cincinnati Children's Hospital Medical Center, Cincinnati, OH 45229, USA

**Keywords:** Primary cilia, Ciliopathies, FGF, C2CD3, Micrognathia, *talpid^2^*

## Abstract

Ciliopathies represent a disease class characterized by a broad range of phenotypes including polycystic kidneys and skeletal anomalies. Ciliopathic skeletal phenotypes are among the most common and most difficult to treat due to a poor understanding of the pathological mechanisms leading to disease. Using an avian model (*talpid^2^*) for a human ciliopathy with both kidney and skeletal anomalies (orofaciodigital syndrome 14), we identified disruptions in the FGF23–PTH axis that resulted in reduced calcium uptake in the developing mandible and subsequent micrognathia. Although pharmacological intervention with the U.S. Food and Drug Administration (FDA)-approved pan-FGFR inhibitor AZD4547 alone rescued expression of the FGF target *SPRY2*, it did not significantly rescue micrognathia. In contrast, treatment with a cocktail of AZD4547 and teriparatide acetate, a PTH agonist and FDA-approved treatment for osteoporosis, resulted in molecular, cellular and phenotypic rescue of ciliopathic micrognathia in *talpid^2^* mutants. Together, these data provide novel insight into pathological molecular mechanisms associated with ciliopathic skeletal phenotypes and a potential therapeutic strategy for a pleiotropic disease class with limited to no treatment options.

## Abstract (precis)

**Summary:** Using an avian model, we report a novel molecular mechanism (disruptions in the FGF23–PTH axis resulting in reduced calcium uptake in the developing mandible) and potential treatment for ciliopathic micrognathia.

## INTRODUCTION

Ciliopathies comprise a growing class of disorders caused by structural or functional disruptions to primary cilia (Goetz and Anderson, 2010; Plotnikova et al., 2009; Reiter and Leroux, 2017). To date, there are ∼35 reported ciliopathies, 180 ciliopathy-associated genes and 250 additional candidate genes (Reiter and Leroux, 2017). Ciliopathies are difficult to treat because they are pleiotropic disorders frequently manifesting in neurological, olfactory, auditory, respiratory, reproductive, excretory and skeletal defects (Goetz and Anderson, 2010; Waters and Beales, 2011). Establishing cellular and molecular etiologies for ciliopathic phenotypes is particularly important because most ciliopathies are life-threatening diseases with limited to no treatment options (Adel Al-Lami et al., 2016).

Ciliopathic skeletal pathologies are among the most difficult of the ciliopathic phenotypes to treat for several reasons. First, these patients frequently have a very limited supply of healthy bone amenable for autograft/allograft treatment. And, even in patients with a supply of healthy bone, grafts frequently suffer from poor efficacy and substantial rejection rates (Holloway et al., 2014; Kahn, 2014). Second, current therapies geared towards inducing bone regeneration [i.e. recombinant bone morphogenic protein (BMP) delivery], likely require functional cilia for signal transduction and have dangerous off-target effects (Holloway et al., 2014). Finally, because very little is known regarding the cellular and molecular mechanisms that contribute to bone dysplasia in ciliopathic patients, generating pharmacological options to treat these conditions has not been possible.

One approach geared towards generating therapeutic strategies for treating ciliopathies is gaining a deeper understanding of molecular mechanisms of cilia-dependent signal transduction. The Hedgehog (Hh) pathway is perhaps the most closely linked and extensively studied pathway relative to ciliary-dependent signal transduction (Briscoe and Therond, 2013; Corbit et al., 2005; Sasai and Briscoe, 2012). Furthermore, the Hh pathway has proven to be very amenable to pharmacological intervention (Lin and Matsui, 2012; Scales and de Sauvage, 2009). Despite these promising opportunities, targeting Hh for the treatment of skeletal phenotypes is problematic due to variable Hh pathway readouts across tissues (i.e. in ciliopathies, some tissues experience a loss of Hh signaling while others experience a gain of Hh signaling) and a lack of Hh-mediated signaling during cellular processes most impacted in skeletal ciliopathies.

Several other pathways essential for skeletogenesis have been purported to utilize the cilium for signal transduction (Horner and Caspary, 2011; Kawata et al., 2021; Kunova Bosakova et al., 2018, 2019; Neugebauer et al., 2009; Wallingford and Mitchell, 2011; Yuan et al., 2019). The fibroblast growth factor (FGF) pathway plays a major role in skeletogenesis, and mutations in certain ciliary proteins result in ectopic expression of genes within the FGF pathway (Kunova Bosakova et al., 2018, 2019; Mina et al., 2007; Tabler et al., 2013; Xie et al., 2020). Moreover, conditions associated with gain-of-function FGF mutations result in phenotypes reminiscent of skeletogenic ciliopathies including decreased bone mass and micrognathia (Kunova Bosakova et al., 2018; Motch Perrine et al., 2019; Zhou et al., 2013). FGF23, a member of the endocrine subfamily of FGF ligands, is essential for bone homeostasis. Expressed in osteocytes and osteoblasts, FGF23 systemically interacts with parathyroid hormone (PTH) to control both bone mineralization and calcium levels throughout the body (Blau and Collins, 2015; Grau et al., 2020; Lu and Feng, 2011; Takashi et al., 2021). Misexpression of FGF23 and PTH results in impaired bone mineralization and osteogenic dysfunction, respectively (Iwasaki-Ishizuka et al., 2005; Lu and Feng, 2011). Interestingly, the FGF23–PTH axis relies heavily on proper kidney function for propagation, as FGF23 signaling induces the secretion of active vitamin D (1,25-D3) from the kidney, which subsequently influences Ca^2+^ levels (Blau and Collins, 2015; Grau et al., 2020; Lu and Feng, 2011; Takashi et al., 2021). Although the impact of impaired FGF23–PTH signaling on bone development has been described, its correlation with skeletal phenotypes observed in ciliopathic mutants has yet to be explored.

Our previous work exploring the etiology of ciliopathic skeletal phenotypes utilized a bona fide avian ciliopathic model called *talpid^2^* (*ta^2^*) (Abbott et al., 1959, 1960). *ta^2^* embryos phenocopy the human skeletal ciliopathy orofaciodigital syndrome 14 (OFD14), presenting with micrognathia, hypoglossia, cleft lip/palate, hypoplastic cerebellar vermis, polydactyly and polycystic kidneys. Genetically, just like human OFD14, *ta^2^* is caused by a mutation in the basal body protein, C2 domain-containing 3 centriole elongation regulator (C2CD3) (Chang et al., 2014). Our previous analyses identified that despite robust proliferation in the precursor population, osteoblasts failed to completely differentiate and mineralize in *ta^2^* embryos. The reduced number of mature osteoblasts was coupled with excessive osteoclast-mediated bone remodeling (Bonatto Paese et al., 2021) that subsequently led to hypoplasia of several skeletal elements of the craniofacial complex. Interestingly, the etiology of bone density disorders (e.g. osteoporosis) was strikingly similar to that observed in *ta^2^* embryos from a phenotypic, cellular and molecular perspective. Phenotypically, patients with bone density disorders frequently had chronic kidney disease (Gal-Moscovici and Sprague, 2007; Tasnim et al., 2021). Cellularly, there was an imbalance of bone resorption and remodeling in patients with bone density disorders (Jevon et al., 2003; Tanaka, 2001; Teitelbaum, 1996). Molecularly, aberrant FGF signaling was described in patients and models with impaired bone mineralization (Kunova Bosakova et al., 2018, 2019; Paul et al., 2019; Tabler et al., 2013). These similarities served as the premise for testing the hypothesis that the FGF23–PTH axis was not only disrupted in skeletal ciliopathies, but also served as a potential therapeutic avenue for the treatment of ciliopathies.

Herein, we propose a novel dual-pronged approach toward alleviating skeletal phenotypes by targeting both the molecular and cellular processes impacted during ciliopathic skeletogenesis. Our data reveal disruptions in FGF signaling, specifically within the FGF23–PTH axis in *ta^2^* embryos. This molecular profile correlates with reduced calcium uptake in the developing mandible and subsequent micrognathia. Treatment with a cocktail of AZD4547, a pan FGFR antagonist, and teriparatide acetate, an osteoporosis drug and PTH agonist, resulted in reduced serum Ca^2+^, increased mineralization and increased size of certain cranial skeletal elements, including the mandible, in *ta^2^* embryos. Together, our data suggest that a targeted approach modulating impaired FGF signaling and excessive bone degradation in ciliopathies, like OFD14, is effective in alleviating ciliopathic skeletal phenotypes.

## RESULTS

### Ciliopathic micrognathia correlates with impaired signaling through the FGF23–PTH axis

**Fig. 1.**
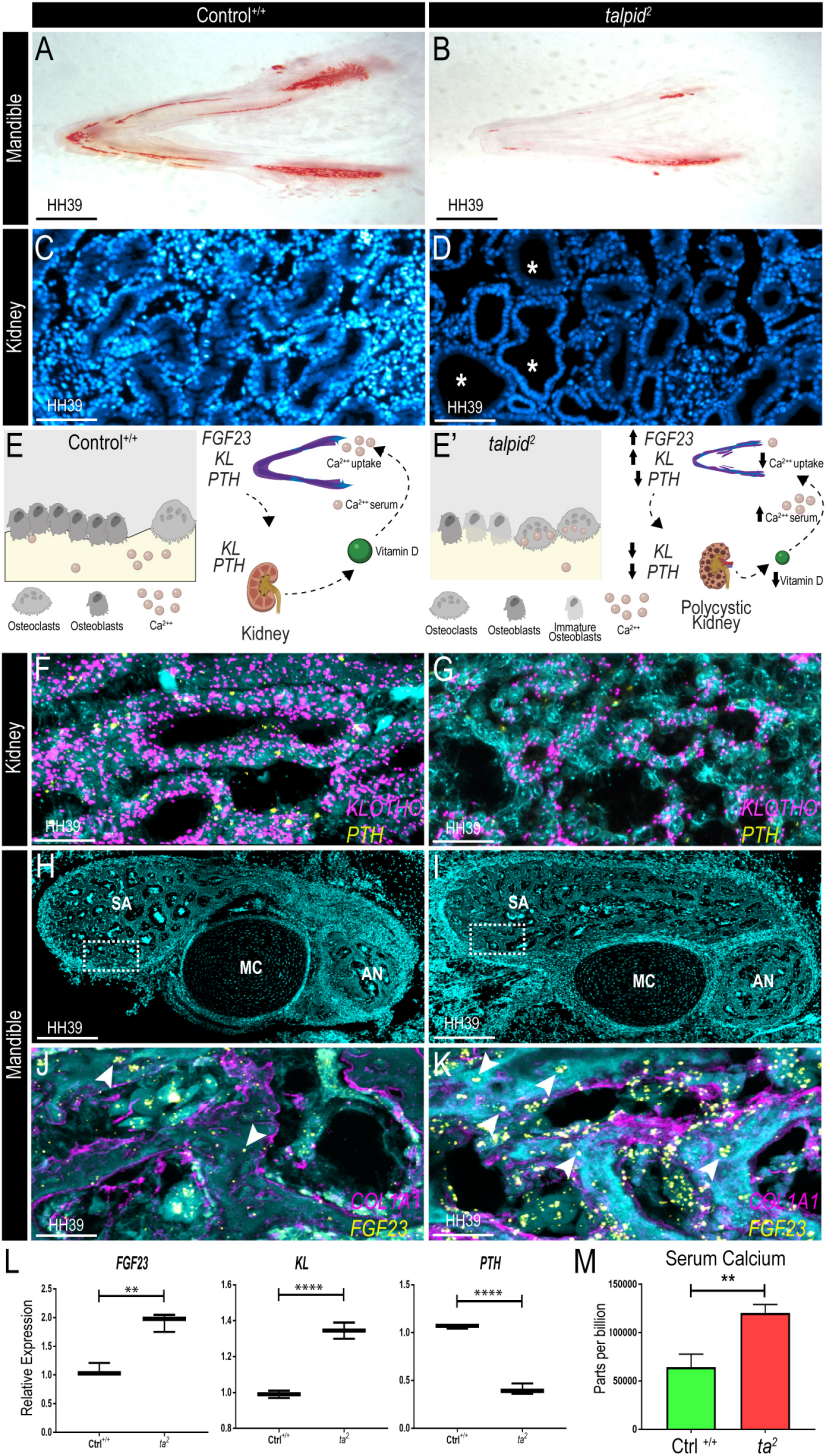
***talpid^2^* (*ta^2^*) mandibles and kidneys have aberrant *FGF23*, *KL* and *PTH* expression.** (A,B) Alizarin Red-stained transverse sections of HH39 control (Ctrl^+/+^) and *ta^2^* mandibles (*n*=3 per group). (C,D) 4′,6-diamidino-2-phenylindole (DAPI)-stained sagittal sections of HH39 Ctrl^+/+^ and *ta^2^* kidneys (white asterisks denote the presence of cystic tubules). (E,E′) Schematic of the FGF23–PTH axis in normal embryonic development (E) and the hypothesized axis in *ta^2^* embryos (E′). (F,G) RNAscope *in situ* hybridization for *KL* (magenta) and *PTH* (yellow) in Ctrl^+/+^ (F) and *ta^2^* (G) HH39 kidney sagittal sections, nuclei counterstained with DAPI (cyan). (H,I) DAPI-stained frontal sections of HH39 Ctrl^+/+^ (H) and *ta^2^* (I) mandible, showing Meckel's cartilage (MC), and the angular (AN) and surangular (SA) bones. White dotted line boxes in H and I indicate the regions analyzed in J and K. (J,K) RNAscope *in situ* hybridization for *FGF23* (yellow) and *COL1A1* (magenta) transcripts in Ctrl^+/+^ (J) and *ta^2^* (K) HH39 mandibular frontal sections, nuclei counterstained with DAPI (cyan) (white arrowheads point to osteocytic expression of *FGF23*). (L) qRT-PCR quantification of *FGF23* (**P=0.0016), *KL* (****P<0.0001) and *PTH* (****P<0.0001) in Ctrl^+/+^ and *ta^2^* HH39 mandibles (*n*=3 per group). (M) Quantification of serum calcium by high-performance liquid chromatography (HPLC) of Ctrl^+/+^ and *ta^2^* embryos (**P=0.0017) at HH39 (*n*=3 per group). Data are mean±s.d. (unpaired two-tailed Student's *t*-test). Scale bars: 1 cm (A,B), 100 µm (C,D), 20 µm (F,G), 100 µm (H,I) and 20 µm (J,K). Schematic created with biorender.com.

Like several ciliopathic models, *ta^2^* embryos present with micrognathia and polycystic kidneys. To characterize the micrognathic phenotype, we utilized Alizarin Red staining, a widely used technique to visualize calcified elements (Elbadawi et al., 1981; Lievremont et al., 1982). Transverse sections of HH39 mandibles [equivalent to mouse embryonic day (E)16.5 and human Carnegie stage (CS)23] revealed reduced Alizarin Red staining in *ta^2^* embryos relative to control (Ctrl^+/+^) embryos (Fig. 1A,B). Our previous data ruled out the possibility that reduced Alizarin Red staining was a consequence of deficiencies in the osteogenic progenitor population (Bonatto Paese et al., 2021), thus suggesting reduced calcium uptake in *ta^2^* mandibles. Concomitantly, frontal sections through HH39 kidneys revealed several cysts within the developing *ta^2^* kidney relative to Ctrl^+/+^ kidney (Fig. 1C,D). Based on the co-presentation of phenotypes, we tested the hypothesis that the FGF23–PTH axis, a signaling pathway that requires kidney function for controlling bone physiology during development, was impaired.

*FGF23* is expressed by osteocytes and osteoblasts and interacts locally with its obligatory receptor klotho (*KL*) and systemically with parathyroid hormone (*PTH*), to regulate bone mineralization and calcium metabolism. These endocrine factors induce the secretion of vitamin D from the kidney. In normal development, vitamin D induces calcium uptake from the serum into bone (Fig. 1E). As per our hypothesis, impaired bone mineralization in the *ta^2^* embryos could be due to aberrant secretion of FGF23 and PTH, and the polycystic phenotype could result in decreased vitamin D production, leading to decreased calcium uptake by the bone and misregulation of *FGF23* and *PTH* expression systemically (Fig. 1E′). To test our hypothesis, we examined the expression of genes within the FGF23–PTH axis in the developing kidney and mandible of HH39 Ctrl^+/+^ and *ta^2^* embryos. RNAscope *in situ* hybridization showed reduced *KL* and *PTH* expression in *ta^2^* compared to Ctrl^+/+^ kidney (Fig. 1F,G). Frontal sections through HH39 mandibles (Fig. 1H,I) revealed that expanded *FGF23* expression was not confined to *COL1A1^+^* osteoblasts; rather it was throughout the medullar region of the bone, where the osteocytes were embedded (Fig. 1J,K). Additionally, quantitative reverse transcription PCR (qRT-PCR) analysis confirmed that *FGF23* and *KL* expression were significantly upregulated, and *PTH* was significantly downregulated, in HH39 *ta^2^* mandibles (Fig. 1L), strongly suggesting aberrant calcium metabolism in *ta^2^* mutants. High-performance liquid chromatography (HPLC) for mineral contents revealed that serum calcium was significantly upregulated in *ta^2^* relative to Ctrl^+/+^ embryos (Fig. 1M). Taken together, our results revealed an imbalance in the FGF23–PTH axis, which was accompanied by reduced calcium uptake in the mandible and subsequently increased calcium in the serum of *ta^2^* embryos. Based on these data, we next explored pharmacological intervention of FGF and PTH activity in *ta^2^* embryos.

### Modulation of the FGF pathway alone does not alleviate ciliopathic micrognathia

**Fig. 2.**
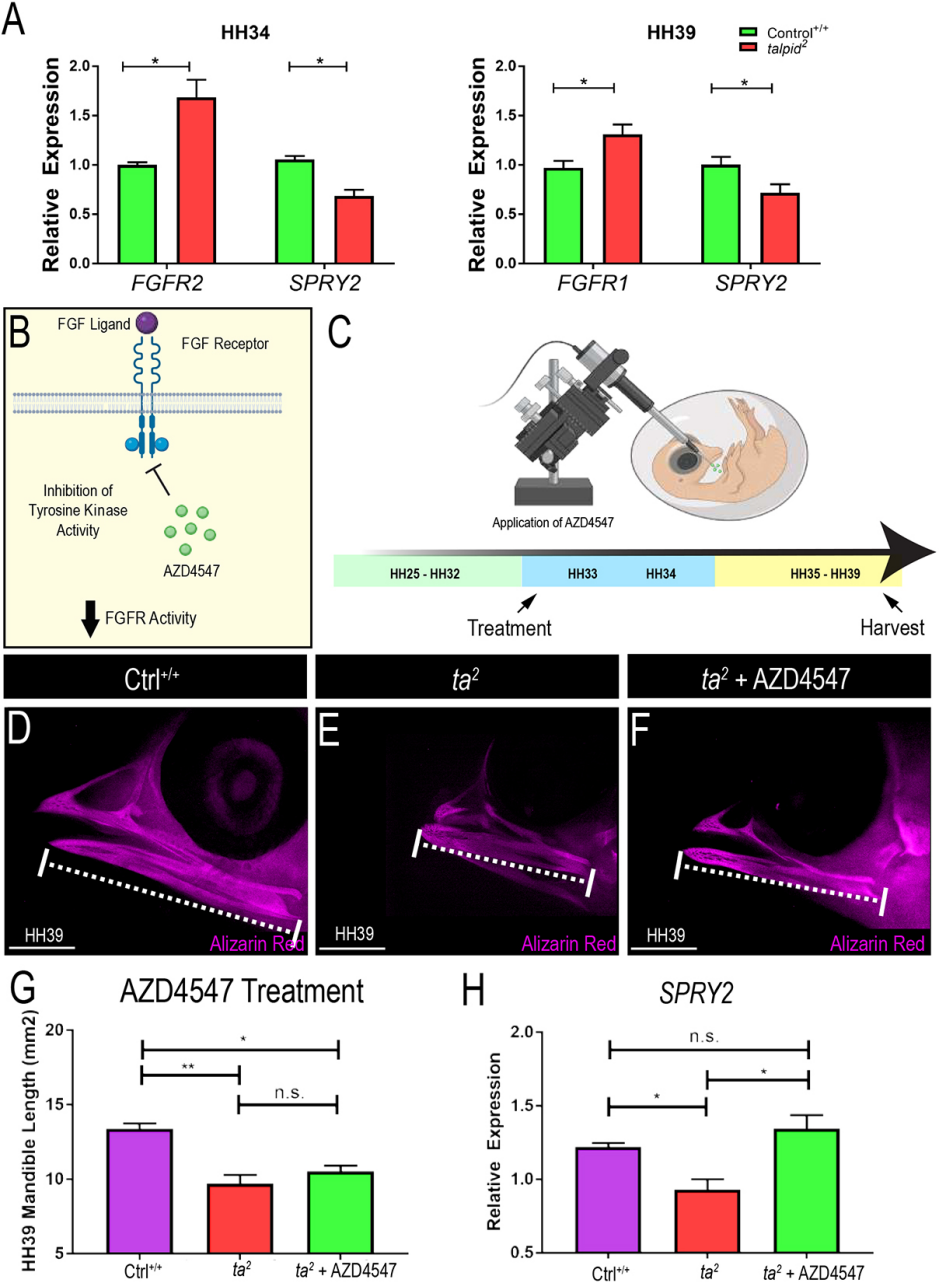
**Overactive FGF signaling can be modulated with AZD4547.** (A) qRT-PCR for *FGFR2* and *SPRY2* at HH34; *FGFR1* and *SPRY2* at HH39 (*P<0.05; *n*=4). (B) Schematic of AZD4547 mechanism. (C) Schematic of the experimental design for AZD4547 treatment. (D-F) Alizarin Red staining in HH39 Ctrl­^+/+^ (D), *ta^2^* (E) and *ta^2^* + AZD4547-treated (F) embryos (*n*=4 for each group). (G) Measurements of the mandibular length of the groups depicted in D-F (*P=0.0174; **P=0.0084). (H) qRT-PCR quantification for *SPRY2* transcripts in the three experimental groups (*P<0.05; *n*=3 per group). Data are mean±s.d. (A) Unpaired one-tailed Student's *t*-test. (G,H) Ordinary one-way ANOVA. n.s., not significant. Scale bars: 2.5 cm. Schematic created with biorender.com.

FGF signaling plays a crucial role in mandibular development (Mina et al., 2007; Takashi et al., 2021; Xie et al., 2020). The master regulator of skeletal development, *RUNX2*, induces the expression of *FGFR2*, and this interaction is responsible for osteoblast proliferation (Kawane et al., 2018). Further, it has been shown that *FGF23* paracrine activity signals exclusively via *FGFR1*, which modulates *FGF23* expression in osteocytes (Takashi et al., 2021; Xiao et al., 2014). We evaluated the expression of *FGFR1* and *FGFR2* during osteoblast maturation (HH34) and bone remodeling (HH39). qRT-PCR revealed a significant upregulation of *FGFR2* and *FGFR1* expression and reduced expression of sprouty 2 (*SPRY2*), a negative regulator of FGF activity, in *ta^2^* embryos at HH34 (equivalent to mouse E12, human CS15) and HH39 (Fig. 2A). Considering these data, we attempted to rescue the micrognathic phenotype in *ta^2^* embryos by pharmacologically inhibiting FGF activity. AZD4547 is a U.S. Food and Drug Administration (FDA)-approved, selective tyrosine kinase inhibitor that targets *FGFR1*, *FGFR2* and *FGFR3* (Fig. 2B). To determine an effective drug dosage in HH33 (equivalent to mouse E11.5, human CS14) embryos a dose–response curve was generated, treating embryos with 10 µl of either 1 μM or 5 μM AZD4547 (Fig. S1A-C). Based on survival rates, 10 µl of 1 μM AZD4547 was utilized and delivered below the chorioallantoic membrane adjacent to the developing mandible (Fig. 2C). At the morphological level, we observed no significant differences between non-injected and injected *ta^2^* embryos (Fig. 2D-G). To determine the efficacy of AZD4547 treatment, expression of the FGF target *SPRY2* was analyzed via qRT-PCR of HH34 mandibular prominence. Interestingly, despite a failure to rescue mandibular length, AZD4547 treatment did rescue *SPRY2* expression to that of Ctrl^+/+^ embryos (Fig. 2H).

Our previous data revealed that increased *FGF23* expression was accompanied by decreased *PTH* expression (Fig. 1). PTH is crucial for the maintenance of calcium homeostasis in the body, acting directly on bone formation and resorption (Silva and Bilezikian, 2015). Thus, we next tested the potential of the PTH agonist teriparatide acetate to rescue ciliopathic micrognathia, using the same experimental design as previously used for AZD4547 delivery (Fig. S2A,B). To determine an effective dosage of teriparatide acetate in HH33 embryos, a dose–response curve was generated, treating embryos with 10 µl of either 1 μM or 10 μM teriparatide acetate. Based on survival rates, 10 µl of 1 μM teriparatide acetate was utilized and delivered as previously described (Fig. S1B,C). The mandibular length was not significantly increased in *ta^2^* embryos treated with teriparatide acetate alone relative to that in untreated *ta^2^* embryos (Fig. S2C-F). Because neither treatment alone significantly improved mandibular length, we next tested a combinatorial treatment.

### AZTeri injection is effective at alleviating ciliopathic micrognathia in *ta^2^* embryos

**Fig. 3.**
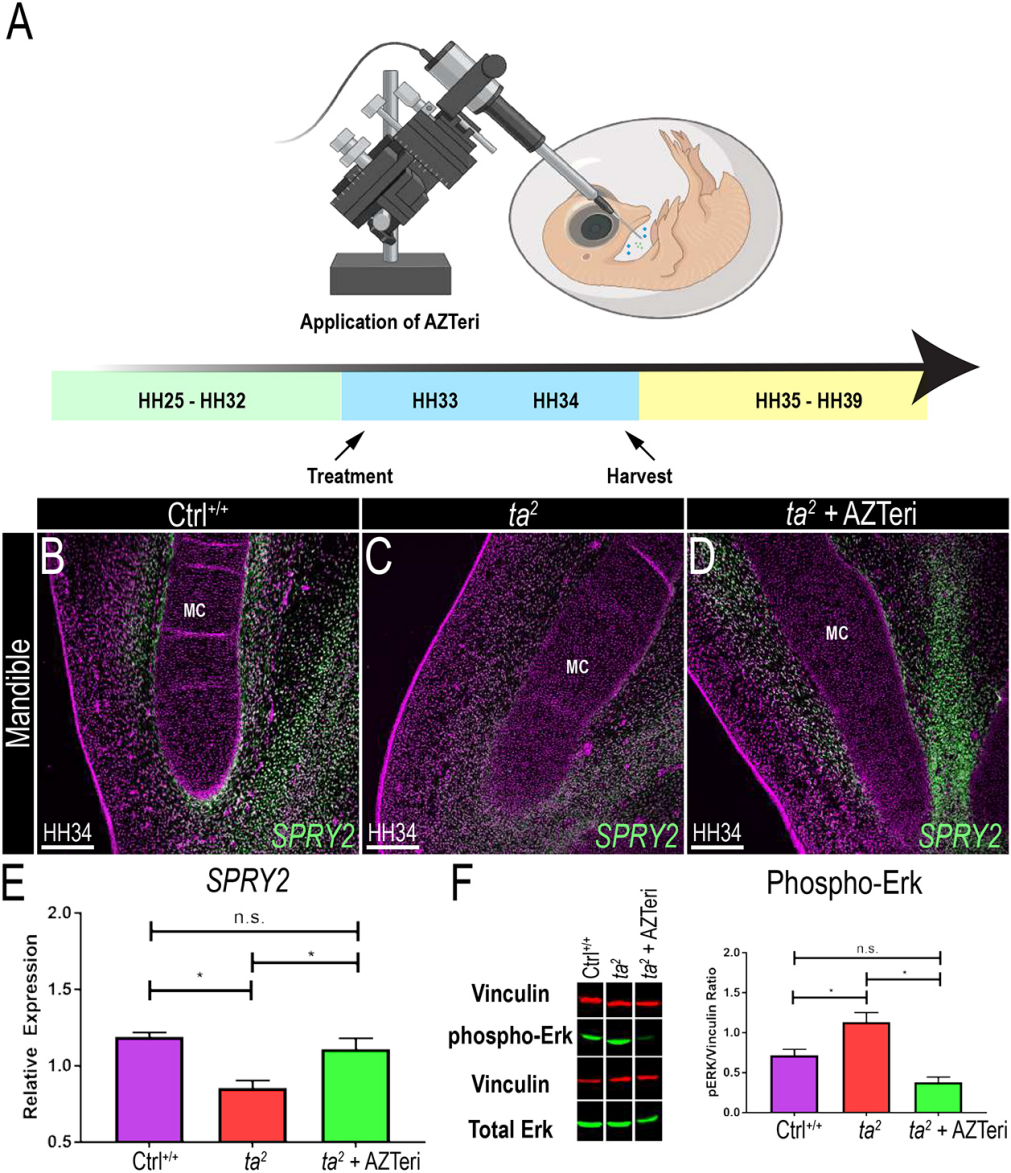
**AZD4547 and teriparatide acetate (AZTeri) treatment in the *ta^2^* mandible.** (A) Schematic of the experimental design for AZTeri treatment. (B-D) RNAscope *in situ* hybridization for *SPRY2* (green) in Ctrl­^+/+^ (B), *ta^2^* (C) and *ta^2^* + AZTeri (D) transverse mandibular sections (*n*=4 per group). (E) qRT-PCR quantification for *SPRY2* transcripts in the three experimental groups (*n*=4 per group). (F) Western blot for phospho-ERK and total ERK, and quantification of phospho-ERK/vinculin ratio in non-injected Ctrl­^+/+^, *ta^2^* and *ta^2^* + AZTeri embryos at HH34 (*n*=3 per group). Nuclei counterstained with DAPI (magenta). MC, Meckel's cartilage. Data are mean±s.d. *P<0.05 (ordinary one-way ANOVA). n.s., not significant. Scale bars: 200 µm. Schematic created with biorender.com.

Given the pleiotropic nature of ciliopathies and the combinatorial cellular mechanism associated with ciliopathic micrognathia, we tested whether treating *ta^2^* embryos with a cocktail of AZD4547 and teriparatide acetate (referred to as AZTeri herein) could yield a significant improvement in ciliopathic micrognathia. The AZTeri cocktail was generated using previously established dosages of individualized AZD4547 and teriparatide acetate treatments (1 μM). HH33 embryos were treated with 10 μl AZTeri and harvested 24 h later at HH34 to assess the efficacy of treatment (Fig. 3A). *SPRY2* expression was expanded in AZTeri-treated *ta^2^* embryos, relative to that in untreated *ta^2^* embryos (Fig. 3B-D). qRT-PCR analysis validated and quantified these data and revealed that *SPRY2* expression in AZTeri-treated *ta^2^* embryos was not significantly different from that observed in untreated Ctrl^+/+^ embryos (Fig. 3E). Western blot analysis further revealed that AZTeri treatment was effective at downregulating MAPK cascade activity. Although there was no change in total ERK (also known as MAPK) levels between untreated and treated Ctrl^+/+^ embryos (Fig. S3), phospho-ERK levels were significantly downregulated in AZTeri-treated *ta^2^* embryos compared to those in the untreated *ta^2^* embryos (Fig. 3F).

**Fig. 4.**
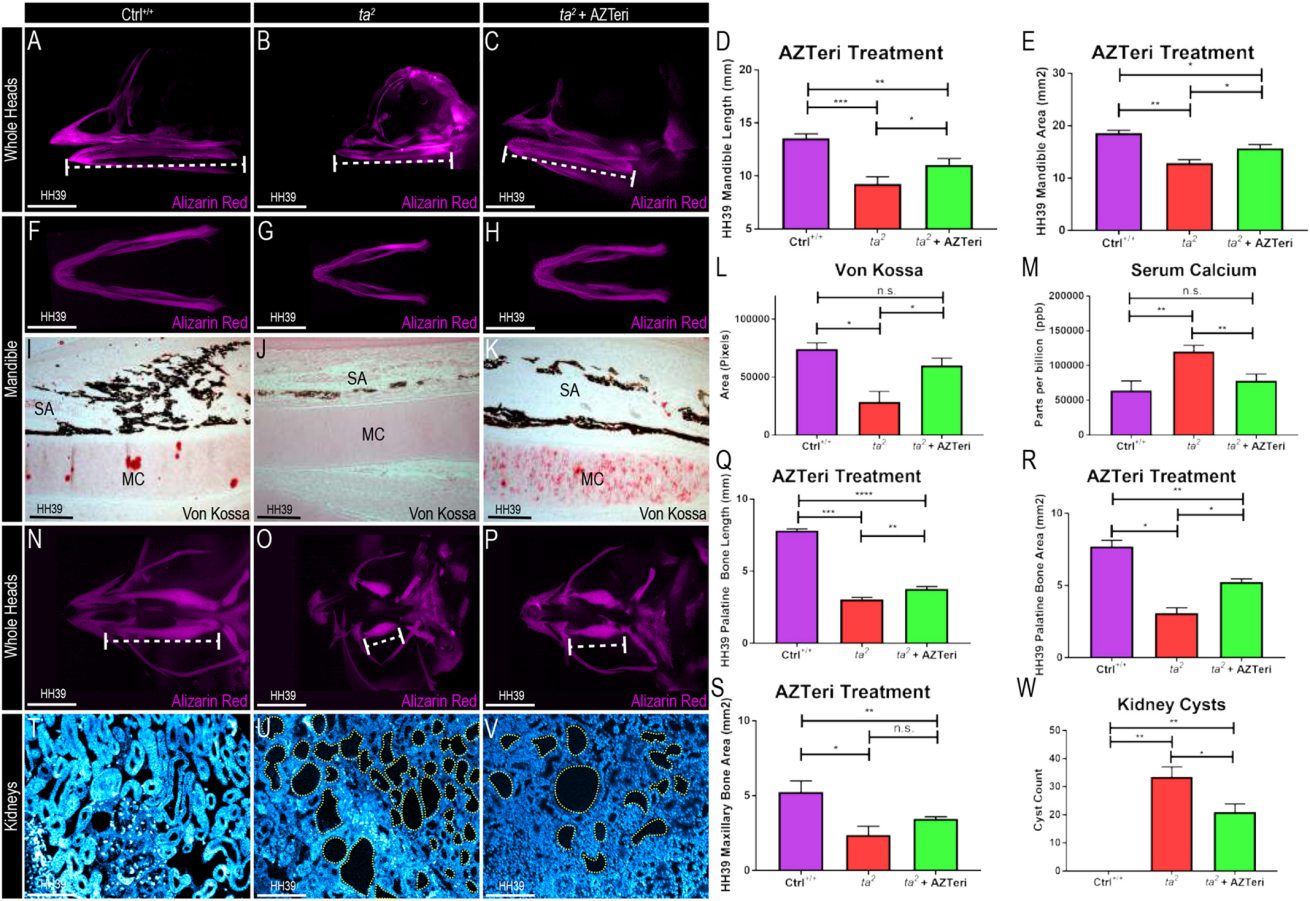
**AZTeri treatment alleviates the micrognathic phenotype in *ta^2^* embryos.** (A-C) Alizarin Red-stained heads at HH39 of Ctrl^+/+^ (A), *ta^2^* (B) and *ta^2^* + AZTeri (C) embryos (*n*=3 for each group). (D) Measurements of the mandibular length of Ctrl^+/+^, *ta^2^* and *ta^2^* + AZTeri embryos (*P=0.0199; **P= 0.0040; ***P=0.0002). (E) Measurements of the mandibular area of Ctrl^+/+^, *ta^2^* and *ta^2^* + AZTeri embryos (Ctrl^+/+^ versus *ta^2^* + AZTeri *P=0.0349; *ta^2^* versus *ta^2^* + AZTeri *P=0.0375; Ctrl^+/+^ versus *ta^2^* **P=0.0052). (F-H) Ventral views of Alizarin Red-stained mandibles at HH39 of Ctrl^+/+^ (F), *ta^2^* (G) and *ta^2^* + AZTeri (H) embryos (*n*=3 for each group). (I-K) Von Kossa staining in transverse sections of Ctrl^+/+^ (I), *ta^2^* (J) and *ta^2^* + AZTeri (K) HH39 mandibles (*n*=4 per group). (L) Area quantification of Von Kossa-stained HH39 mandibular sections (*P<0.05). (M) Quantification of serum calcium by HPLC of Ctrl^+/+^, *ta^2^* and *ta^2^* + AZTeri HH39 embryos (**P<0.05; *n*=3 per group). (N-P) Ventral views of Alizarin Red-stained heads at HH39 of Ctrl^+/+^ (N), *ta^2^* (O) and *ta^2^* + AZTeri (P) embryos (*n*=3 for each group). (Q) Measurements of the palatine bone length of Ctrl^+/+^, *ta^2^* and *ta^2^* + AZTeri embryos (*n*=3 for each group) (**P=0.0266; ***P=0.0001; ****P<0.0001). (R) Measurements of the palatine bone area of Ctrl^+/+^, *ta^2^* and *ta^2^* + AZTeri embryos (*n*=3 for each group) (Ctrl^+/+^ versus *ta^2^* + AZTeri *P=0.0122; *ta^2^* versus *ta^2^* + AZTeri *P=0.0175; Ctrl^+/+^ versus *ta^2^* **P=0.0020). (S) Measurements of the maxillary bone area of Ctrl^+/+^, *ta^2^* and *ta^2^* + AZTeri embryos (*n*=3 for each group) (*P=0.0162; **P=0.0016). (T-V) DAPI-stained sagittal sections of Ctrl^+/+^ (T), *ta^2^* (U) and *ta^2^* + AZTeri (V) embryos (cysts are denoted by yellow dotted lines) (*n*=3 per group). (W) Quantification of cyst numbers in kidneys of Ctrl^+/+^, *ta^2^* and *ta^2^* + AZTeri HH39 embryos (*n*=3 per group) (*P=0.0351; **P=0.0022; **P=0.0082). MC, Meckel's cartilage; SA, surangular bone. Data are mean±s.d. (ordinary one-way ANOVA). n.s., not significant. Scale bars: 2.5 cm (A-C), 2.5 cm (F-H), 200 µm (I-K), 2.5 cm (N-P), 20 µm (T-V).

To test the potential of AZTeri as a therapeutic agent for skeletal ciliopathies, HH33 embryos were treated with 10 µl AZTeri and harvested at HH39. AZTeri-treated *ta^2^* embryos demonstrated a significant increase in mandibular length and area compared to untreated *ta^2^* embryos (Fig. 4A-H). Von Kossa staining revealed increased mandibular calcification in AZTeri-treated *ta^2^* embryos compared to untreated *ta^2^* embryos (Fig. 4I-L), and the increased amounts of mandibular calcification correlated with decreased levels of serum calcium (Fig. 4M). Furthermore, the therapeutic benefits of AZTeri were not limited to the mandible, as palatine and maxillary bones of treated *ta^2^* embryos were also increased in size, albeit not significantly in the case of the maxilla (Fig. 4N-S). To evaluate the systemic potential of AZTeri treatments, the number of cysts in treated and untreated *ta^2^* embryos was analyzed. Although cysts were still present in the AZTeri-treated *ta^2^* embryos, they were significantly reduced in number compared to that in the untreated *ta^2^* embryos (Fig. 4T-W). Finally, to characterize the molecular and cellular impact of AZTeri treatment, we performed RNAscope *in situ* hybridization and tartrate-resistant acid phosphatase (TRAP) staining in HH39 mandibles. Although AZTeri treatment did not significantly restore *PTH* expression to the level observed in Ctrl^+/+^ embryos, it did significantly reduce *SPP1* expression compared to that in the untreated *ta^2^* embryos (Fig. S4A-H). This molecular profile, coupled with reduced TRAP staining, confirmed that bone remodeling was reduced in AZTeri-treated *ta^2^* embryos (Fig. S4I-K). Taken together, these results demonstrated the potential of AZTeri treatment for ciliopathic micrognathia.

## DISCUSSION

Herein, we present a potential avenue for the pharmacological intervention of ciliopathic skeletal phenotypes. Utilizing the *ta^2^* avian mutant as a model for a human ciliopathy, we identified disruptions in the FGF23–PTH signaling axis concomitant with decreased bone mineralization and increased serum calcium. These data, in concert with our previous reports that excessive bone resorption contributed to ciliopathic micrognathia (Bonatto Paese et al., 2021), informed our hypothesis that a treatment that simultaneously targeted FGF signaling and bone resorption would rescue micrognathia in *ta^2^* embryos. These findings support a potential drug-based therapeutic option for human ciliopathy patients.

Avians are an exquisite model for pharmacological testing due to *in ovo* embryonic accessibly, low cost and an abundant number of embryos (Hebert et al., 2005; Hodges, 1946; Karnofsky and Lacon, 1964; Kue et al., 2015; Robel, 1993; Vidal, 1953; Zosen et al., 2021), despite drug efficacy and metabolism being distinct from those in mammals. Several drugs currently used in preclinical cancer trials or treatments were initially tested on avian embryos (Bueker and Platner, 1956; Karnofsky and Lacon, 1964; Kue et al., 2015; Ryley, 1968; Zuniga et al., 2003). Although *in ovo* screens have provided a wealth of information on toxicity and off-target effects, the lack of avian models for human disease has prevented more robust usage of the egg as a tool for testing pharmacological agents in human health research.

The *ta^2^* is perfectly suited for such studies. First, it phenocopies human ciliopathies on both a genetic and biochemical level and survives well into development. Second, because most ciliopathic models are early embryonic lethal, murine conditional knockout models are commonly used to study molecular mechanisms. Although this is effective in examining a ciliopathic insult on one tissue, it fails to consider the pleiotropic nature of ciliopathies as they present in human patients. As such, the *ta^2^* represents a unique and powerful model that is not only easily accessible but also highly representative of a human ciliopathy (Bonatto Paese et al., 2021; Chang et al., 2014; Schock et al., 2015).

One of the most common skeletal phenotypes associated with ciliopathies is micrognathia. Micrognathia significantly impacts a patient's ability to breathe, eat and speak. Treatment options for micrognathia are limited. Surgical procedures, like distraction osteogenesis, are highly invasive, and the poor quality of the bone in ciliopathy patients makes treatment like this less effective (Abramson et al., 2013; Breik et al., 2016; Holloway et al., 2014; Kahn, 2014; Perlyn et al., 2002; Tomonari et al., 2017). To eliminate the need for surgical intervention, pharmacological treatments for micrognathia have been explored. Drug treatments for osteoporosis, broadly defined as “an imbalance between bone formation and bone resorption”, were seen as strong candidates for treatment (Bodenner et al., 2007). Mechanistically, this description is very similar to the pathology observed in the *ta^2^* mandibles (Bonatto Paese et al., 2021). Bisphosphonates represent potent inhibitors of bone resorption that are FDA approved for the treatment of osteoporosis. In an avian model, bisphosphonate treatment significantly elongated the mandible (Ealba et al., 2015). Despite the efficacy of bisphosphonate treatment in avians, treatment in humans has proven less effective and has been associated with the development of bisphosphonate-related osteonecrosis of the jaw (BRONJ) (Eckert et al., 2007; Rayman et al., 2009). Thus, additional experiments focusing on alternative pharmacological treatments for micrognathia are necessary.

Teriparatide acetate, a component of the AZTeri treatment used herein, represents another FDA-approved treatment for osteoporosis. Teriparatide acetate effectively reduces bone resorption and has shown promising results in phase 4 trials (Leder, 2017). It has been successfully used for the treatment of BRONJ (Chopra and Malhan, 2020; Dos Santos Ferreira et al., 2021; Kwon and Kim, 2016; Sim et al., 2020; Yu and Su, 2020), and reduced serum calcium levels and improved bone integrity in osteoporosis and hypoparathyroidism patients (Gutierrez-Cerecedo et al., 2016; Satterwhite et al., 2010). Considering the variable efficacy and side effects in human patients, it will be important to carefully examine other osteoporosis-approved drugs (Denosumab, etc.) for the treatment of ciliopathic skeletal phenotypes (Tsai et al., 2019).

In addition to targeting the cellular process of bone resorption with, we also hypothesized that treating excessive FGF activity would prove necessary for the treatment of micrognathia. Previous results revealed an association between ciliopathies and FGF syndromes; however, the association was specifically between FGF signaling and the onset of maxillary phenotypes, such as high arched palate (Tabler et al., 2013). Mandibular ciliopathic phenotypes, on the other hand, have been more commonly associated with aberrant Hh or Wnt signaling (Elliott et al., 2018; Millington et al., 2017; Zhang et al., 2011). Although much of the data on FGF and mandibular development focus on an early patterning role of FGF8 (Mina et al., 2007; Shigetani et al., 2000; Terao et al., 2011; Zhou et al., 2013), FGF23 plays an important role later in skeletal development by modulating parathyroid hormone and calcium signaling (Blau and Collins, 2015; Lu and Feng, 2011). As Hh and Wnt signaling have numerous roles throughout the embryo at this stage of skeletogenesis, focusing specifically on FGF23 signaling may prove to be the most targeted mode of treatment for pleiotropic diseases, like ciliopathies, with skeletal phenotypes. Despite our results, it will be important to continue careful examination of off-target effects of AZD4547, as FGF signaling has numerous essential roles in the body. Our treatment strategy was a singular application well after several essential developmental milestones (e.g. gastrulation, neural tube closure, facial prominences patterning). As such, we potentially avoid many off-target effects and target the specific processes impacted in skeletal ciliopathies: osteoblast maturation and bone remodeling.

Calcium signaling plays a pivotal role during bone development, and depleted calcium uptake is the main cause of conditions such as osteoporosis and rickets (Monsen, 1989). There is no consensus as to whether the primary cilium plays a major role in calcium signaling (Delaine-Smith et al., 2014; Delling et al., 2013, 2016; Hoey et al., 2012; Lee et al., 2015; Malone et al., 2007; Saternos et al., 2020), yet our results support a systemic role for cilia in the differentiation of osteoblasts (Bonatto Paese et al., 2021). It is possible that the role of cilia in calcium uptake may vary between tissues (e.g. node versus osteoblast), temporally during development, or between chemosensory and mechanosensory cilia. More detailed experiments will need to be done to definitively determine the relationship between the cilium and calcium uptake in the developing mandible.

In summary, our work proposes a novel molecular mechanism and treatment strategy for ciliopathic micrognathia using a cocktail of FDA-approved drugs. This treatment not only improved mandibular length and mineralization, but also partially restored the size of the palatine bone and decreased the number of cysts in the kidney. As a complete rescue of micrognathia may be optimistic at this time, a realistic goal for this treatment option is to restore the mandible to a length that alleviates the need for repeated, invasive surgeries and allows patients a better quality of life.

## MATERIALS AND METHODS

### Embryo collection and genotyping

Fertilized Ctrl^+/+^ and *ta^2^* eggs were purchased from the University of California, Davis. Eggs were incubated at 38.8°C in a rocking incubator with humidity control. Staging followed the Hamburger–Hamilton staging system, and genotyping was performed as previously described (Bonatto Paese et al., 2021; Hamburger and Hamilton, 1951). Unless noted otherwise in figure legends, every experiment utilized five embryos for each experimental group.

### Skeletal staining

Samples were incubated in 0.005% Alizarin Red S (Sigma-Aldrich, A5533) in 1% KOH for 3 h at room temperature and cleared in 1% KOH. Once cleared, samples were incubated in glycerol:KOH 1% (50:50) solution. For imaging and long-term storage, samples were kept in 100% glycerol. Stained specimens were imaged using a Leica M165 FC stereo microscope system.

### qRT-PCR

RNA was extracted using TRIzol reagent (Invitrogen), and cDNA was synthesized using SuperScript III (Invitrogen). HH39 mandibles were first frozen with liquid nitrogen and ground using a mortar and pestle to ensure homogenous extraction. SYBR Green Supermix (Bio-Rad) and a Quant6 Applied Biosytems qPCR machine were used to perform qRT-PCR. All the genes were normalized to *GAPDH* expression. Negative controls were performed by omitting the cDNA in the mixture. The level of expression for each gene was calculated using the 2^−ΔΔCq^ method (Livak and Schmittgen, 2001). Unpaired one-tailed Student's *t*-test was used for statistical analysis. *P*<0.05 was determined to be significant.

### RNAscope *in situ* hybridization

RNAscope *in situ* hybridization was carried out as previously described (Bonatto Paese et al., 2021). The transcripts used in this study – *FGF23* [Advanced Cell Diagnostics (ACD) 1002831], PTH (ACD 1003861), *SPP1* (ACD 571601) and *SPRY2* (ACD 1086991) – were detected using a RNAscope Multiplex Fluorescent V2 kit as per the manufacturer's instructions. Both sections and wholemount samples were imaged using a Nikon A1 LUN-V inverted microscope system.

### Embryonic treatment

Three mixes were utilized in this study: AZD4547 (Selleck Chem, S2801) was diluted to 1 µM in 4% dimethyl sulfoxide+30% polyethylene glycol 300+5% Tween 80+ddH_2_O; teriparatide acetate (Selleck Chem, P1033) was diluted to 1 µM in ddH_2_O; AZTeri was a mix of 1 µM AZD4547 and 1 µM teriparatide acetate diluted in ddH_2_O. Embryos were treated at HH33 via applying 10 µl of the drugs under the chorioallantoic membrane immediately adjacent to the mandible. Embryos were then incubated without shaking in the incubator. Wholemount heads were dissected at either HH34 or HH39 and processed for further analysis.

### Analysis of serum calcium content

Blood (100 µl) from the vitelline vein of HH39 embryos was collected on ice with microcapillaries, weighed and sent for processing by the R. Marshall Wilson Mass Spectrometry facility at the University of Cincinnati. Inductively coupled plasma mass spectrometry with HPLC was utilized.

### Histological analysis

Hematoxylin and Eosin (H&E) staining was performed using standard protocols. For calcium deposit analysis, 7 µm transverse sections of HH39 mandibles were used with the Von Kossa Stain Kit (Calcium Stain) (Abcam, ab150687), following the manufacturer’s instructions. TRAP staining was performed on 8 μm-thick transverse sections of decalcified HH39 mandibles using an Acid Phosphatase Leukocyte (TRAP) Kit (Sigma-Aldrich, 387A) following the manufacturer's protocol. Automated cyst quantification was performed with the software CystAnalyser, with the settings for kidney cysts (Cordido et al., 2020).

### Western blotting

Embryos were injected at HH33, and mandibles were dissected at HH34 for processing. Collected tissue was sonicated in cold RIPA buffer (50 mM Tris-HCl, pH 7.4, 1% NP-40, 0.25% sodium deoxycholate, 150 mM NaCl, 1 mM EDTA) containing protease and phosphatase inhibitors (ThermoFisher Scientific, 78440). The protein extract was collected after 10 min full-speed centrifugation at 4°C at 17,000 ***g***. Twenty micrograms of protein from each embryo were used for western blotting, with the following primary and secondary antibodies: anti-ERK1/2 (Cell Signaling Technology, 9101S, 1:1000), anti-phospho-p44/42 MAPK (ERK1/2) (Novus Biologicals, NB110-96887, 1:1000), anti-vinculin (Santa Cruz Biotechnology, sc-73614, 1:2000), IRDye^®^ 800CW donkey anti-rabbit IgG (LICOR, 926-32213, 1:2000), IRDye^®^ 680RD donkey anti-mouse IgG (LICOR, 925-68072, 1:2000). Images were taken by LICOR Odyssey^®^ DLx. Densitometry was done by ImageJ.

### Statistical methods

Unpaired *t*-tests (two groups) or one-way ANOVA (three and four groups) were used in comparisons for statistical analysis between groups. *P*<0.05 was considered significant for two-tailed analysis.

## Supplementary Material

Supplementary information
