# Supplementary material for: Pharmacological intervention of the FGF–PTH axis as a potential therapeutic for craniofacial ciliopathies
Source: Dis Model Mech. 2022 Aug 16;15(8):dmm049611. doi: 10.1242/dmm.049611 (PMC9403750; doi:10.1242/dmm.049611)
Supplement: Supplementary information [file dmm-15-049611-s1.pdf]

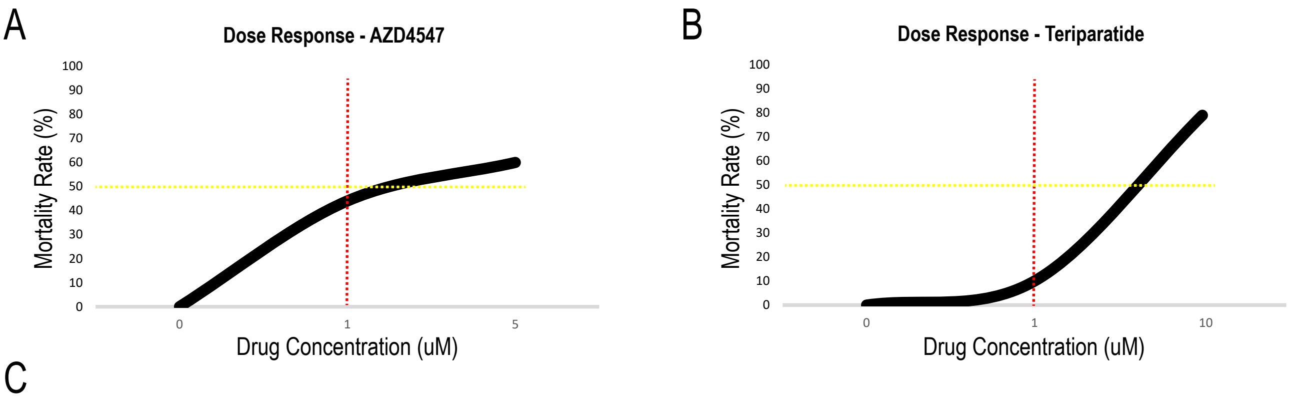

C

AZD4547			
**Drug concentration (µM)**	**0**	**1**	**5**
**Mortality (%)**	0	44	60
**Live Embryos**		14	15
**Total Embryos**		25	37

Teriparatide			
**Drug concentration (µM)**	**0**	**1**	**10**
**Mortality (%)**	0	10	79
**Live Embryos**		20	7
**Total Embryos**		22	32

Fig. S1. (A) Dose response curve of AZD4547 treatment. X-axis represents drug concentration (uM) and Y-axis represents the mortality rate (%) of total embryos treated. (B) Dose response curve of Teriparatide Acetate treatment. X-axis represents the drug concentration (uM) and Y-axis represents the mortality rate (%) of total embryos treated. Yellow-dashed line shows the 50% mortality rate, and the red-dashed line represents the chosen concentration. (C) Table containing the number of embryos treated for each drug concentration of AZD4547 and Teriparatide Acetate.

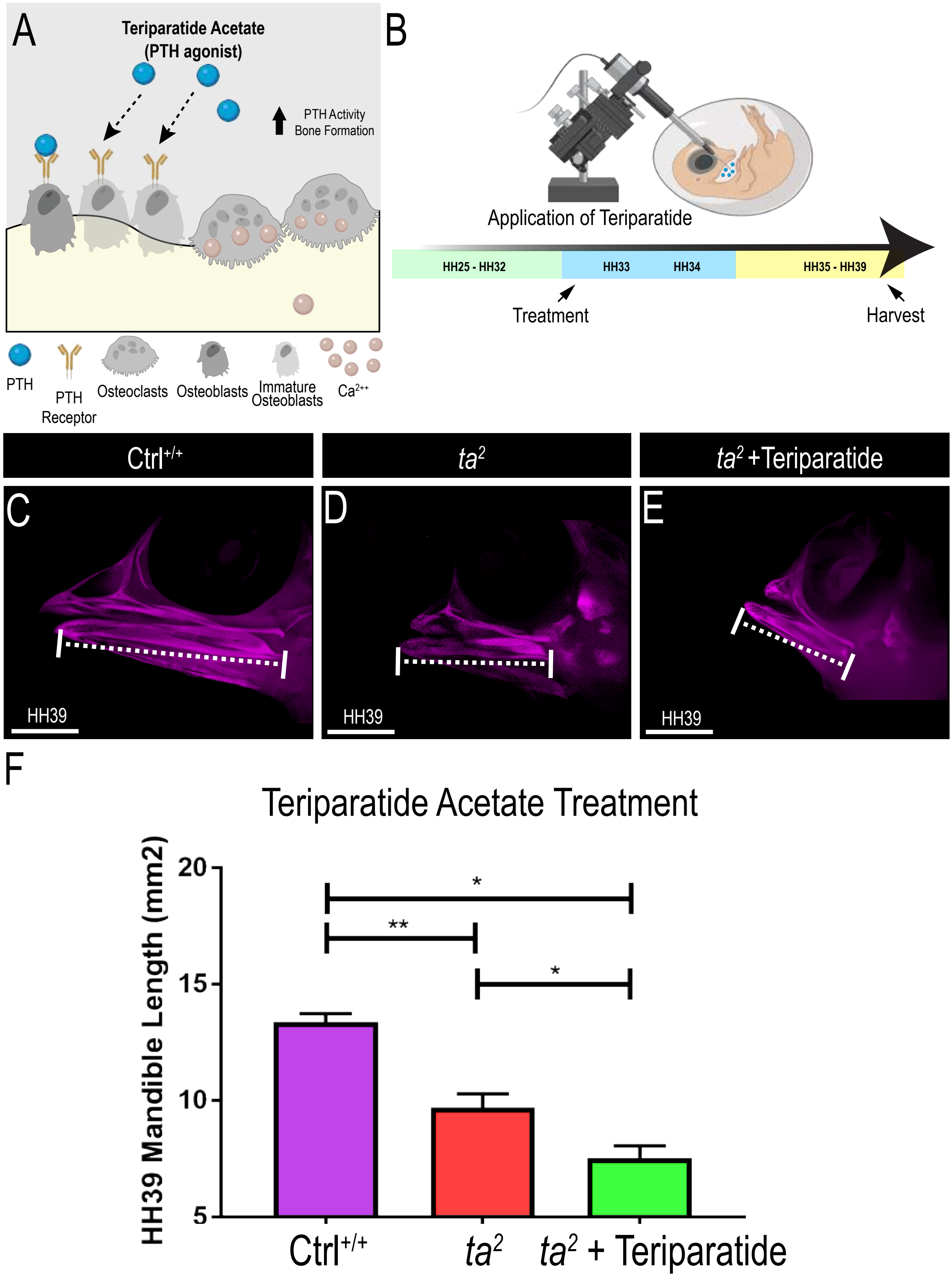

Fig. S2. (A) Schematic of the mechanism of action for Teriparatide Acetate. (B) Experimental design for Teriparatide Acetate treatment. (C-E) HH39 Alizarin Red stained heads of Ctrl+/+, ta2 and ta2 + Teriparatide Acetate embryos (n=3 for each group). (F) Measurements of the mandibular length of the groups depicted in C-E ($p > 0.05$). Data are mean±s.d. (ordinary one-way ANOVA). n.s, not significant. Scale bars: (C-E) 2.5cm. (Schematic created with BioRender.com).

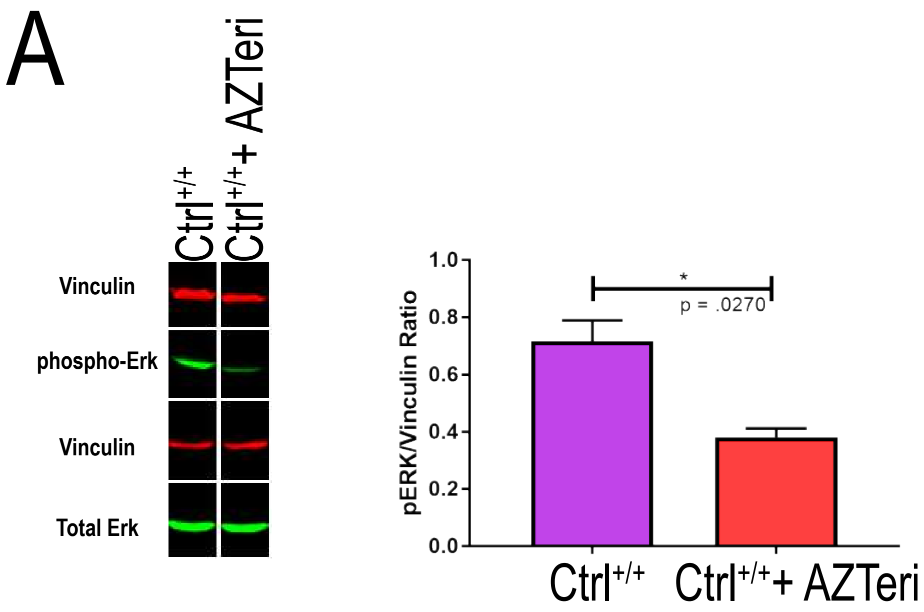

Fig. S3. (A) Western blot for phosphorylated-ERK and total ERK, and quantification of pERK/Vinculin ratio in Ctrl+/+ and Ctrl+/+ + AZTeri embryos at HH34 (n=3 per group). Data are mean±s.d. *P<0.05 (unpaired one-tailed Student's t-test).

Disease Models & Mechanisms • Supplementary information

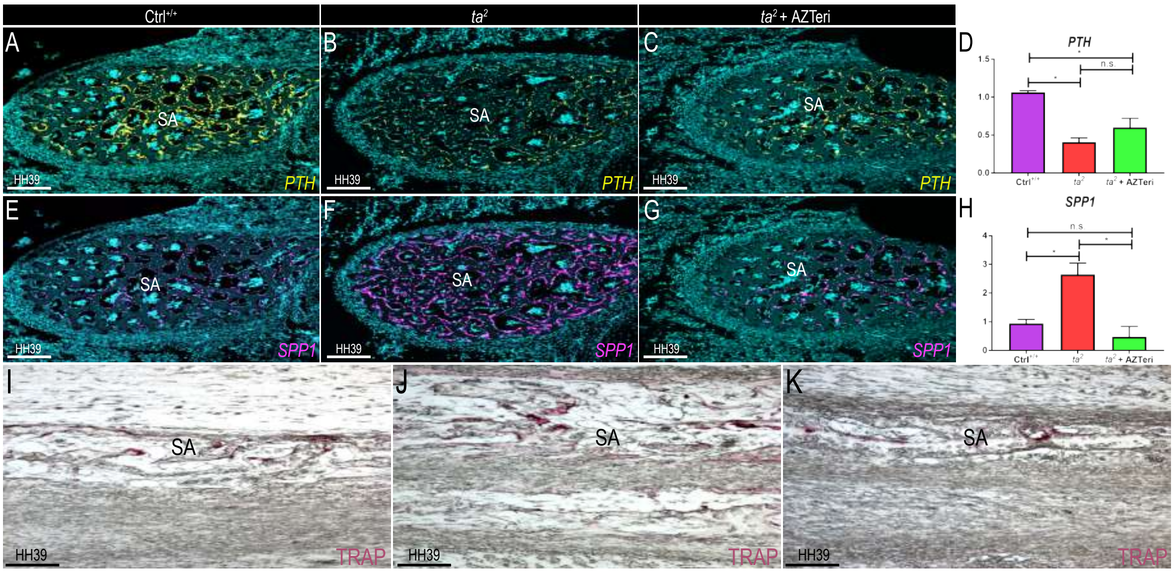

Fig. S4. (A-C) RNAscope in situ hybridization for PTH (yellow) in Ctrl$^{+/+}$, *ta2* and *ta2* + AZTeri HH39 mandibular frontal sections (n=3 per group). (D) qRT-PCR quantification for *PTH* transcripts in the three experimental groups (n=4 per group). (E-G) RNAscope in situ hybridization for *SPP1* transcripts (magenta) in Ctrl$^{+/+}$, *ta2* and *ta2* + AZTeri HH39 mandibular frontal sections (n=3 per group). (H) qRT-PCR quantification for *SPP1* transcripts in the three experimental groups (n=4 per group). (I-K) TRAP staining in transverse sections of Ctrl$^{+/+}$, *ta2* and *ta2* + AZTeri HH39 mandibles (n= 4 per group). Scale bars: (I-K) 20µm.

Disease Models & Mechanisms • Supplementary information
